# Crystal structures of main protease (M^pro^) mutants of SARS-CoV-2 variants bound to PF-07304814

**DOI:** 10.1186/s43556-023-00134-2

**Published:** 2023-08-03

**Authors:** Haihai Jiang, Xiaofang Zou, Pei Zeng, Xiangyi Zeng, Xuelan Zhou, Jie Wang, Jin Zhang, Jian Li

**Affiliations:** 1 School of Basic Medical Sciences, Nanchang University, Nanchang, 330031 China; 2 College of Pharmaceutical Sciences, Gannan Medical University, Ganzhou, 341000 China; 3 Shenzhen Crystalo Biopharmaceutical Co., Ltd., Shenzhen, 518118 China; 4 Jiangxi Jmerry Biopharmaceutical Co., Ltd., Ganzhou, 341000 China

**Keywords:** SARS-CoV-2, Variant, Main protease, Inhibitor, PF-07304814

## Abstract

There is an urgent need to develop effective antiviral drugs to prevent the viral infection caused by constantly circulating SARS-CoV-2 as well as its variants. The main protease (M^pro^) of SARS-CoV-2 is a salient enzyme that plays a vital role in viral replication and serves as a fascinating therapeutic target. PF-07304814 is a covalent inhibitor targeting SARS-CoV-2 M^pro^ with favorable inhibition potency and drug-like properties, thus making it a promising drug candidate for the treatment of COVID-19. We previously solved the structure of PF-07304814 in complex with SARS-CoV-2 M^pro^. However, the binding modes of PF-07304814 with M^pro^s from evolving SARS-CoV-2 variants is under-determined. In the current study, we expressed six M^pro^ mutants (G15S, K90R, M49I, S46F, V186F, and Y54C) that have been identified in Omicron variants including the recently emerged XBB.1.16 subvariant and solved the crystal structures of PF-07304814 bound to M^pro^ mutants. Structural analysis provided insight into the key molecular determinants responsible for the interaction between PF-07304814 and these mutant M^pro^s. Patterns for PF-07304814 to bind with these investigated M^pro^ mutants and the wild-type M^pro^ are generally similar but with some differences as revealed by detailed structural comparison. Structural insights presented in this study will inform the development of novel drugs against SARS-CoV-2 and the possible conformation changes of M^pro^ mutants when bound to an inhibitor.

## Introduction

In late December of 2019, a novel coronavirus was detected in Wuhan city, situated in Hubei Province of central China [1, 2]. In February 2020, severe acute respiratory syndrome coronavirus 2 (SARS-CoV-2) was designated as the name of this newly emerging coronavirus by the International Committee on Taxonomy of Viruses (ICTV). SARS-CoV-2 is known as the pathogenic agent responsible for the coronavirus disease-19 (COVID-19) [3]. Being highly transmissible, SARS-CoV-2 has spread quickly to the entire world and posed a serious threat to global public health [4, 5]. As of April 26, 2023, over 764 million confirmed cases and more than 6.9 million deaths have been reported worldwide, with the numbers still increasing (https://covid19.who.int/). The impressive commitment made by the biomedical research community led to rapid development of several safe and effective vaccines [6–9], which plays an important role in reducing the rates of infection, hospitalization, and mortality. However, the continuing emergence of SARS-CoV-2 variants highlights that the battle against COVID-19 is far from over. Five variants have been characterized by the World Health Organization (WHO) as variants of concern (VOCs), namely Alpha (B.1.1.7), Beta (B.1.351), Gamma (P.1), Delta (B.1.617.2), and Omicron (B.1.1.529), and several variants have been designated as variants of interest (VOIs), including Lambda (C.37) and Kappa (B.1.617.1). Omicron is currently the most prevalent SARS-CoV-2 variant and has evolved into many sub-variants with high immune evasion ability, including BA.5, BF.7, BQ.1, XBB.1.5, and the newly emerged XBB.1.16 [10–14], which may associate with the reduced effectiveness of available vaccines. Therefore, effective and curative treatment measures are still urgently needed to combat COVID-19.

Development of small molecules that exert antiviral efficacies against SARS-CoV-2 infection by inhibiting its main protease (M^pro^), a vital enzyme in the viral life cycle, continues to be a relevant trend in searching for COVID-19 treatments [15]. M^pro^ is able to cleave the bio-synthesized viral polyprotein 1ab (pp1ab) at 11 specific sites to produce a variety of mature non-structural proteins necessary for viral replication and transcription [16, 17]. In most cases, this enzyme has a recognition sequence of (Leu-Gln)-(Ser-Ala-Gly), with the bond between glutamine and serine being the cleavage site [18, 19]. Such an enzymatic cleavage specificity is absent in humans, which indicates the absence of toxicity of potential M^pro^ inhibitors [18, 19]. Thus, inhibitors targeting the main protease can effectively impede the infection of SARS-CoV-2 and represent promising antiviral drug candidates.

**Fig. 1:**
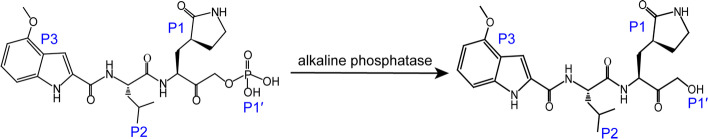
SARS-CoV-2 M^pro^ inhibitor PF-07304814. PF-07304814 is a phosphate ester prodrug of PF-00835231 that is rapidly metabolized into PF-00835231 by alkaline phosphatase, The subsites of PF-07304814 and PF-00835231 are indicated

In the last three years, various M^pro^ inhibitors have been discovered by using the high-throughput screen or structure-based drug design [20]. Among these, PF-07304814, also named lufotrelvir, is a highly soluble phosphate prodrug of PF-00835231 and a first in class 3CL inhibitor to treat SARS-CoV-2 infection [21]. It is developed by Pfizer and need to be administered by the intravenous route. When administrated, PF-07304814 will be cleaved by ubiquitous human alkaline phosphatase and metabolized into its active form, namely PF-00835231 (Fig. 1), which exhibits potent antiviral efficacy against SARS-CoV-2 as well as favorable absorption, distribution, metabolism, excretion (ADME) profiles [21]. The crystal structure of SARS-CoV-2 M^pro^ bound to PF-00835231 has been determined [22]. Recently, our group also solved the crystal structure of SARS-CoV-2 M^pro^ bound to the drug candidate PF-07304814 (PDB ID 7VVP) and illustrated the molecular mechanism for interaction [23]. The carbonyl carbons of hydroxymethyl ketone (HMK) warheads of PF-00835231 and PF-07304814 form irreversible covalent bonds with the sulfur atom of M^pro^ active-site cysteine (Cys145). These data provide structural basis and support that PF-00835231 as well as PF-07304814 is a valuable scaffold for developing effective drugs to treat COVID-19.

To date, multiple mutations in the main proteases of emerging SARS-CoV-2 variants are identified [24, 25], which may perturb the interaction network between PF-00835231 and SARS-CoV-2 M^pro^, thus affecting its potency. PF-07304814 has the similar molecular structure with its active form. Determining the interacting details between PF-07304814 and M^pro^ mutants will provide valuable information on drug resistance to PF-00835231 and therapeutic implication for COVID-19. In this study, we solved the crystal structures of PF-07304814 in complex with several M^pro^ mutants, each carrying a previously reported single amino acid substitution, and revealed the structural basis for their interactions. The results provide structural insights for understanding the possible interaction differences between PF-07304814 and various mutant M^pro^s, and will add to develope more effective drugs to treat viral infection caused by SARS-CoV-2 as well as its variants.

## Results

### Structure determination of M^pro^ mutants-PF-07304814 complexes

A total of six previously reported mutations (G15S, K90R, M49I, S46F, V186F, and Y54C) are included in this investigation. The occurrence rates for G15S, K90R, M49I, S46F, and V186F mutations among 15,476,050 sequenced SARS-CoV-2 M^pro^ genes in the Global Initiative on Sharing All Influenza Data (GISAID) (accessed on April 27, 2023) are 0.19%, 1.34%, 0.015%, 0.025%, and 0.016%, respectively, while only 13 reports are available regarding the occurrence of Y54C mutation. All these six mutations are identified in the wildly contagious Omicron variants. G15S and K90R mutations, previously dominant in Lambda and Gamma variants, respectively, are also found in the recently emerged XBB.1.16 subvariant. In order to figure out the interaction details between PF-07304814 and these six mutant M^pro^s, the co-crystallization method was used to determine the structures of M^pro^ mutants bound to PF-07304814. All M^pro^ mutants were expressed in *E. Coli* cells, purified to near homogeneity, and then incubated with excessive PF-07304814 (Fig. 1). The prepared samples were successfully crystallized and the crystal structures of PF-07304814 in complex with these M^pro^ mutants were solved to 2.29-Å (G15S), 1.95-Å (S46F), 2.05-Å (M49I), 1.75-Å (Y54C), 1.97-Å (K90R), and 1.70-Å (V186F) resolution, respectively. All these complex structures are in space group *P*12_1_1, which is different with that (*P*2_1_2_1_2_1_) of wild-type M^pro^-PF07304814 complex [23]. Data collection and refinement statistics are summarized in Table 1.

**Table 1:** Data collection and refinement statistics

	SARS-CoV-2 M^pro^ G15S-PF-07304814	SARS-CoV-2 M^pro ^S46F-PF-07304814	SARS-CoV-2 M^pro^ M49I-PF-07304814	SARS-CoV-2 M^pro^ Y54C-PF-07304814	SARS-CoV-2 M^pro^ K90R-PF-07304814	SARS-CoV-2 M^pro^ V186F-PF-07304814
**PDB Code**	8HVU	8HVV	8HVW	8HVX	8HVY	8HVZ
**Data collection**						
Space group	*P*12_1_1	*P*12_1_1	*P*12_1_1	*P*12_1_1	*P*12_1_1	*P*12_1_1
a, b, c(Å)	55.70, 99.26, 59.9	55.29, 98.65, 59.50	55.13, 99.23, 59.64	55.16, 99.35, 59.80	54.69, 98.56, 58.86	55.33, 99.15, 59.54
α, β, γ(°)	90.00, 108.26, 90.00	90.00, 108.18, 90.00	90.00, 107.95, 90.00	90.00, 107.51, 90.00	90.00, 107.50, 90.00	90.00, 108.00, 90.00
Wavelength(Å)	0.97918	0.97918	0.97918	0.97918	0.97918	0.97918
Resolution(Å)^a^	2.29 (2.35–2.2)	1.95 (2.07–1.95)	2.05 (2.10–2.05)	1.75 (1.85–1.75)	1.97 (2.07–1.97)	1.70 (1.79–1.70)
Total reflections	182,238	207,373	225,654	371,530	256,472	386,686
Unique reflections^a^	27,534	41,127	37,920	61,385	38,245	58,251
* R*_merge_(%)^a^	10.4 (85.6)	5.8 (34.0)	7.0 (94.6)	4.5 (59.1)	6.3 (67.6)	4.2 (62.5)
Mean I/σ(I)^a^	9.6/2.0	15.3/4.4	14.4/2.0	14.5/2.8	16.2/2.7	21.0/2.6
Completeness(%)^a^	98.6(99.9)	93.5(99.3)	99.0(97.1)	99.7(99.6)	90.4(86.8)	86.2(97.0)
Redundancy	6.6(6.5)	5.0(4.3)	6.0(4.3)	6.1(5.9)	6.7(6.7)	6.6(6.0)
**Refinement**						
Resolution (Å)	56.88–2.29	35.96–1.95	49.62–2.05	49.67–1.75	48.78–1.97	52.62–1.70
* R*_work_/*R*_free_^b^	21.20/25.51	19.95/23.87	21.06/24.81	21.59/23.64	21.55/24.83	21.49/23.84
Atoms	4616	4657	4601	4377	4589	4575
Mean temperature factor (Å^2^)	40.6	34.9	39.5	34.4	42.8	34.2
Bond lengths (Å)	0.009	0.007	0.007	0.007	0.007	0.006
Bond angles (°)	1.156	0.873	0.861	0.911	0.852	0.864
Ramachandran plot						
Favored (%)	97.29	97.96	97.81	98.05	97.64	98.12
Allowed (%)	2.71	2.04	2.19	1.95	2.36	1.88
Outliers (%)	0	0	0	0	0	0
Molprobity	1.58	1.25	1.10	1.10	1.15	1.06

*R*_merge_ = Σ_*hkl*_Σ_*i*_|*I*_*i*_(*hkl*)**-**˂*I*(*hkl*)˃| / Σ_*hkl*_Σ_*i*_*I*_*i*_(*hkl*), where ˂I(*hkl*)˃ is the mean intensity of a set of equivalent reflections

*R*_work_ = Σ_*hkl*_||*F*_obs_|-|*F*_calc_|| / Σ_*hkl*_|*F*_obs_|, where *F*_obs_ and *F*_calc_ are observed and calculated structure factors, respectively

^a^The values in parentheses are for the outermost shell

^b^*R*_free_ is the *R*_work_ based on 5% of the data excluded from the refinement

### Overall structure of M^pro^ mutants bound to PF-07304814

**Fig. 2:**
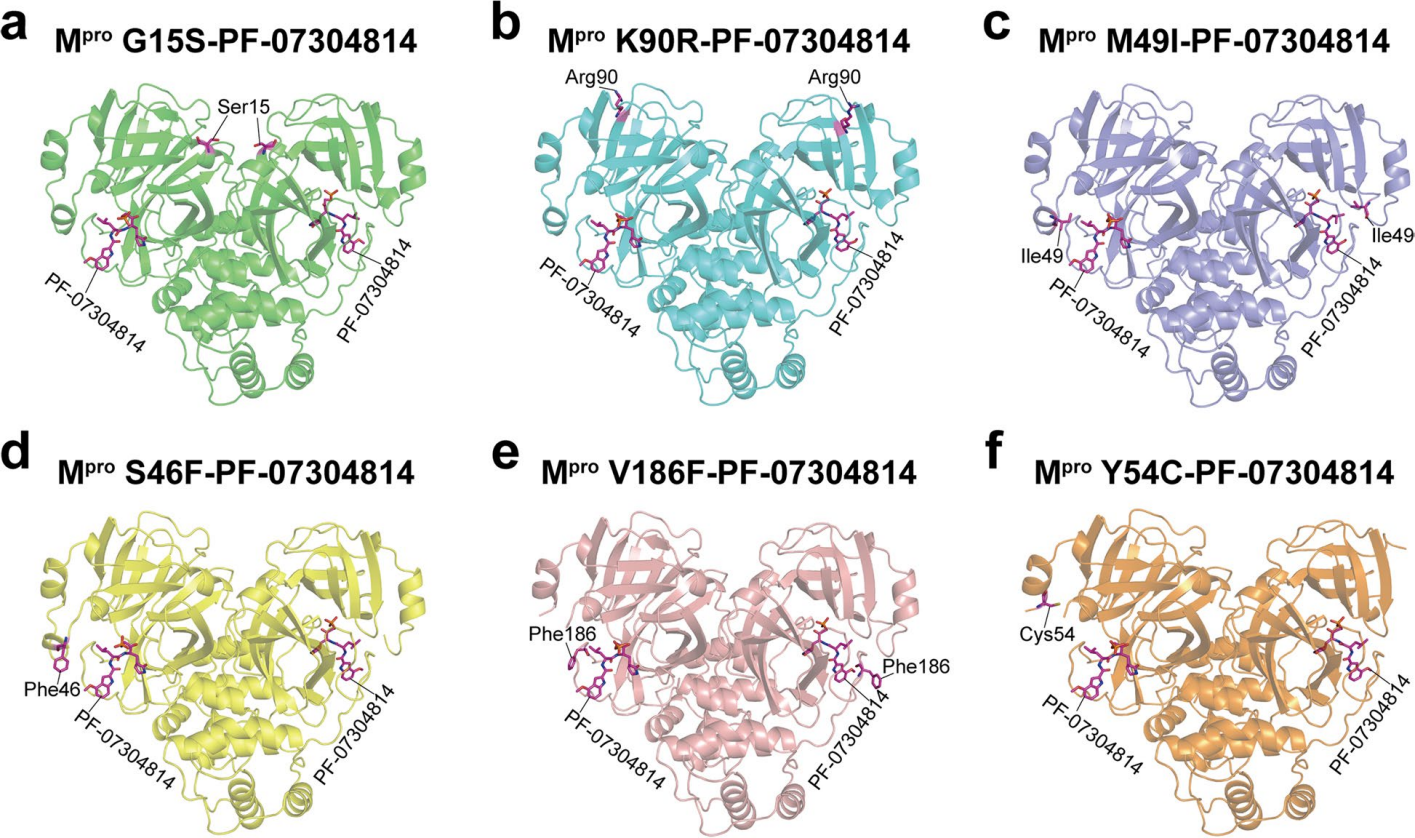
Structural overview of SARS-CoV-2 M^pro^ mutants in complex with PF-07304814. **a** SARS-CoV-2 M^pro^ G15S (green) in complex with PF-07304814 (magentas). **b** SARS-CoV-2 M^pro^ K90R (cyan) in complex with PF-07304814 (magentas). **c** PF-07304814 (magentas) bound to SARS-CoV-2 M^pro^ M49I (slateblue). **d** PF-07304814 (magentas) in complex with SARS-CoV-2 M^pro^ S46F (yellow). **e** PF-07304814 (magentas) bound to SARS-CoV-2 M^pro^ V186F (salmon). **f** PF-07304814 (magentas) bound to SARS-CoV-2 M^pro^ Y54C (orange). The SARS-CoV-2 M^pro^ mutants are shown as cartoons, while PF-07304814 molecules and mutated residues are displayed as sticks

**Fig. 3:**
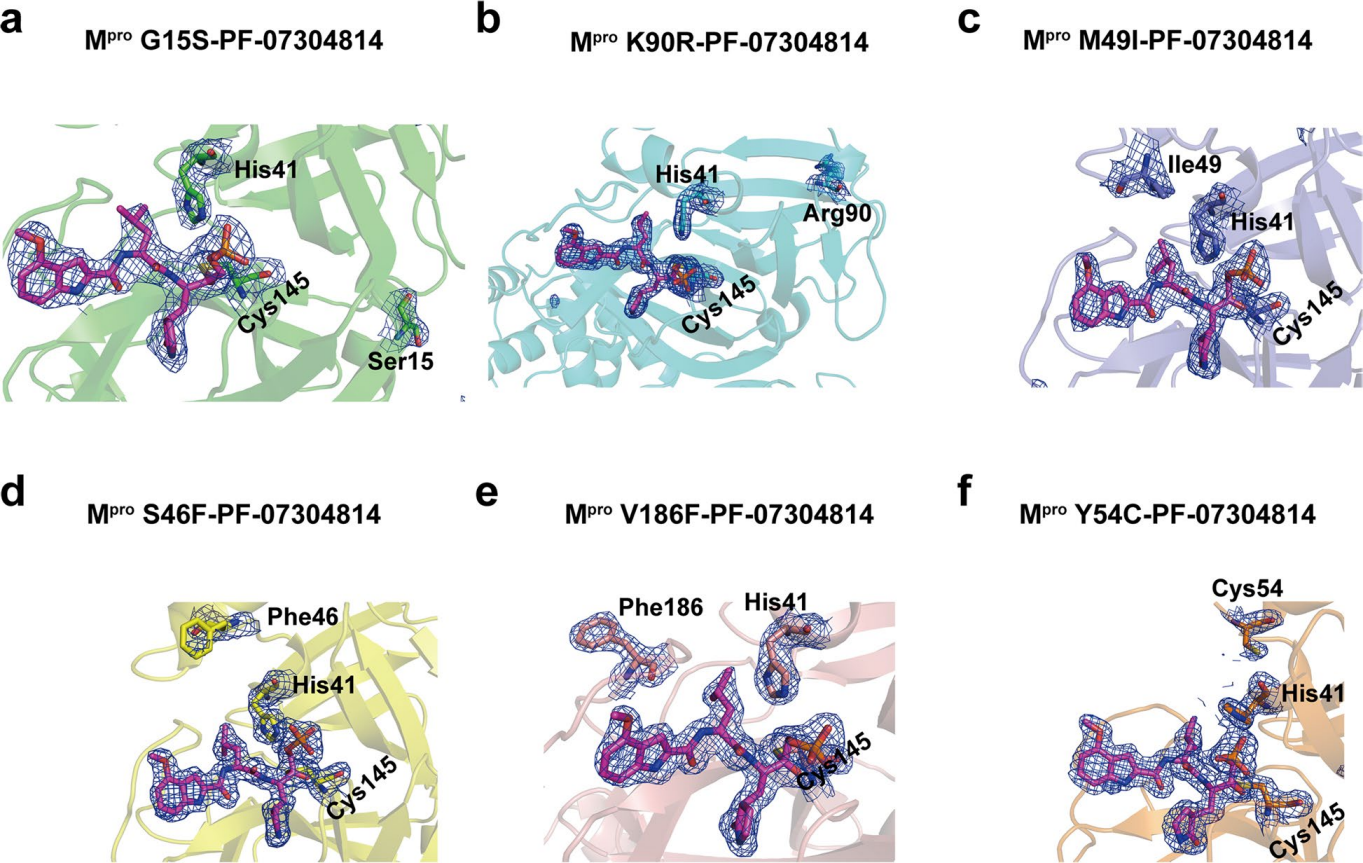
The electron density maps of ligands, catalytic dyad residues, and mutant residues in M^pro^ mutants-PF-07304814 complexes. **a-f** The 2*Fo-Fc* density map (1.0σ) of PF-07304814 (magentas) molecules bound to M^pro^ G15S (**a**, green), K90R (**b**, cyan), M49I (**c**, slateblue), S46F (**d**, yellow), V186F (**e**, salmon), and Y54C (**f**, orange) are shown as a blue mesh. The ligands, catalytic dyad residues, and mutant residues are shown as sticks

In these solved complex structures, each mutant M^pro^ is displayed as a dimer, which is exactly the form with enzymatic activity. Like the wild-type SARS-CoV-2 M^pro^, each protomer of the dimeric M^pro^ mutants contains three major domains (domain I-III). Domain I resides between residues 10 through 99 and forms an anti-parallel β sheet. Domain II is located between residues 100 through 184 and is composed of anti-parallel β sheet structures. Domain III starts at the residue 201 and ends at the residue 303, which is consisting of several ɑ helices and connects to domain II by a long loop region (residues 185–200). All the SARS-CoV-2 M^pro^ mutants bind with two PF-07304814 molecules. PF-07304814 was found to insert into the active sites of mutant M^pro^s (Fig. 2), which is situated in the cleft between domains I and II. We then extracted the electron density maps of the ligand PF-07304814, the catalytic residues (including His41 and Cys145), and the mutant residues. Cys145 is a nucleophilic reagent in the hydrolysis process of SARS-CoV-2 M^pro^ mutant, and the binding of PF-07304814 will inhibit its hydrolysis reaction. As unambiguously shown by the electron density maps, the carbonyl carbon of the hydroxymethylketone (HMK) warhead of PF-07304814 covalently interact with the sulfur atom of Cys145 in SARS-CoV-2 M^pro^ mutants (Fig. 3). Two of these mutations (G15S and K90R) are distal to the substrate binding site of SARS-CoV-2 M^pro^, while the other four mutations (M49I, S46F, V186F, and Y54C) are located in or nearby the substrate binding site (Figs. 2 and 3). However, the electron densities for mutant residues S46F and Y54C could only be traced in one protomer of the M^pro^ (Fig. 2). The quality of the fitting of two PF-07304814 molecules in the electron density maps has been evaluated by two parameters, namely the real-space R-factor (RSR) and the real-space correlation coefficient (RSCC). Calculated RSR values for the ligand 1 and ligand 2 range from 0.10 to 0.15 and from 0.08 to 0.17, respectively, while RSSC values for the ligand 1 and ligand 2 range from 0.87 to 0.93 and from 0.90 to 0.93, respectively (Table 2).

**Table 2:** RSR and RSCC values for ligands in the SARS-CoV-2 M^pro^ mutants-PF-07304814 complexes

Complexes	RSR (real-space R-factor)		RSCC (real-space correlation coefficient)	
	Ligand 1	Ligand 2	Ligand 1	Ligand 2
G15S-PF−07304814	0.15	0.17	0.92	0.91
K90R-PF−07304814	0.14	0.12	0.90	0.90
M49I-PF−07304814	0.13	0.12	0.91	0.92
S46F-PF−07304814	0.13	0.11	0.92	0.92
V186F-PF−07304814	0.10	0.08	0.93	0.93
Y54C-PF−07304814	0.14	0.10	0.87	0.93

### The binding energy between PF-07304814 and M^pro^ mutants

A molecular docking analysis was performed to characterize the binding energy between PF-07304814 and the six SARS-CoV-2 M^pro^ mutants as well as the wild-type SARS-CoV-2 M^pro^. The binding energy values in kcal/mol are described in Table 3. As shown in Table 3, the binding energy between wild-type M^pro^ and PF-07304814 is -9.2 kcal/mol. In comparison, PF-07304814 shows higher binding energy with the six M^pro^ mutants. Among these, PF-07304814 shows the least binding energy with V186F M^pro^, but shows the highest binding energy with M49I M^pro^. The binding energy values between PF-07304814 and these M^pro^ mutants are − 8.9 (V186F), -7.4 (G15S), -7.4 (K90R), -6.8 (S46F), -6.6 (Y54C), and − 6.3 (M49I) kcal/mol. These interaction energy data may reflect the strength of the enzyme-ligand complex.

**Table 3:** The binding energies between PF-07304814 and SARS-CoV-2 M^pro^ mutants

M^pro^s	wild-type	G15S	K90R	M49I	S46F	V186F	Y54C
Binding energy with ligand (kcal/mol)	−9.2	−7.4	−7.4	−6.3	−6.8	−8.9	−6.6

### Comparison of binding modes of PF-07304814 with different M^pro^ mutants

**Fig. 4:**
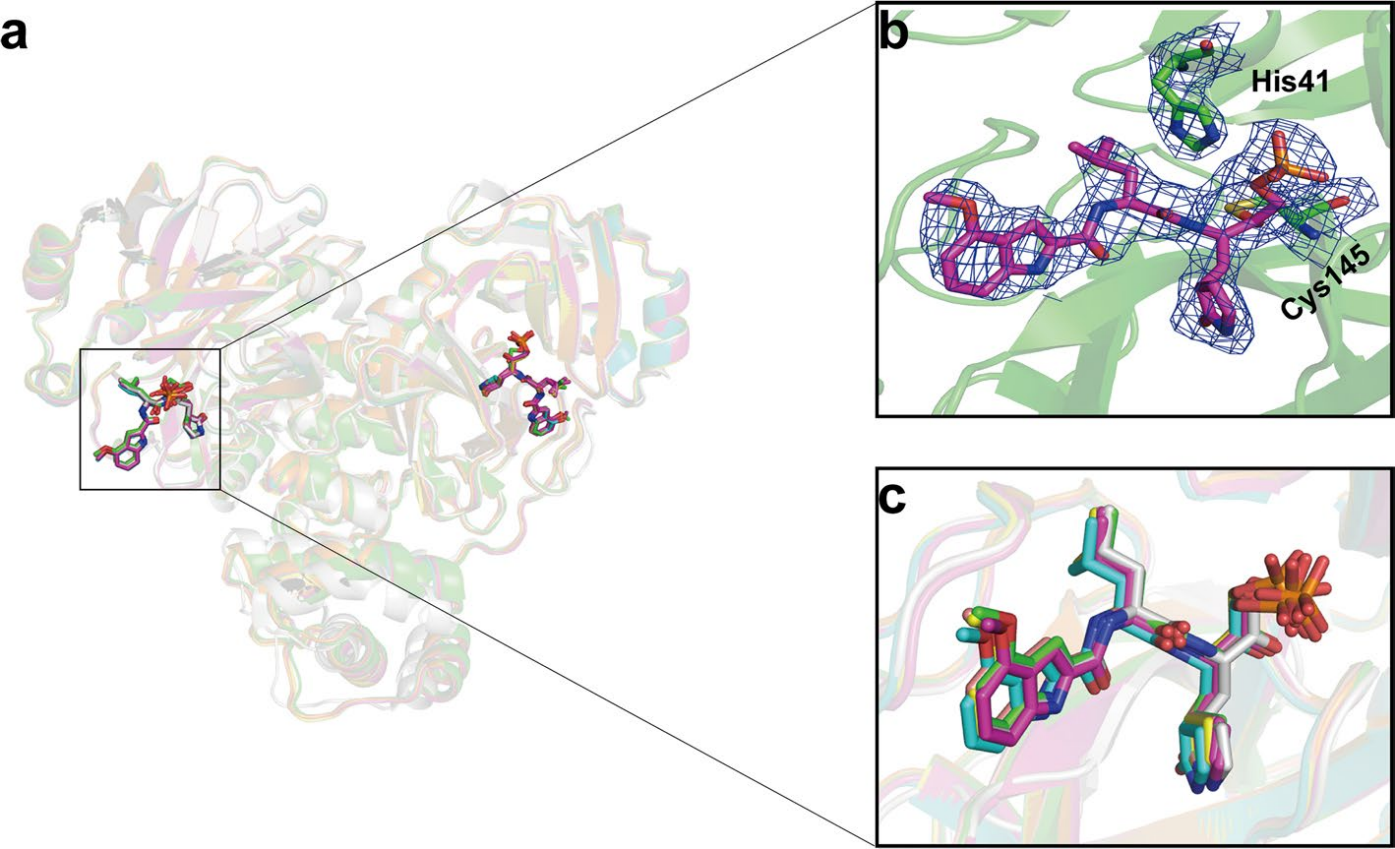
Structural comparison of SARS-CoV-2 M^pro^-PF-07304814 complexes. **a** Overview of structural superposition of wild-type M^pro^-PF-07304814 complex (gray) and M^pro^ G15S-PF-07304814 complex (green), M^pro^ K90R-PF-07304814 complex (cyan), M^pro^ M49I -PF-07304814 complex (slateblue), M^pro^ S46F-PF-07304814 complex (yellow), M^pro^ V186F-PF-07304814 complex (salmon), and M^pro^ Y54C-PF-07304814 complex (orange). The SARS-CoV-2 M^pro^ and its mutants are shown as cartoons, while PF-07304814 molecules are displayed as sticks. **b** The representative electronic density map of the ligands. **c** A enlarged view of structural superpositions

To determine whether conformation of different M^pro^ mutants is changed when bound to PF-07304814, we superposed these six structures with wild-type M^pro^-PF-07304814 complex. The root mean square deviation (RMSD) values of equivalent Cα positions range from 0.695 to 0.808 Å. The results showed that the binding patterns of PF-07304814 are not significantly perturbed by these substitutions on SARS-CoV-2 M^pro^ (Fig. 4). Indeed, two substitutions, namely G15S and K90R, are far from the binding site of PF-07304814 and have little impact on the interaction between PF-07304814 and M^pro^. Though four other substitutions, namely S46F, M49I, Y54C, and V186F, situated nearby the binding site of PF-07304814, no significant changes were found in the orientation of PF-07304814.

### Detailed interaction between PF-07304814 and different M^pro^ mutants

**Fig. 5:**
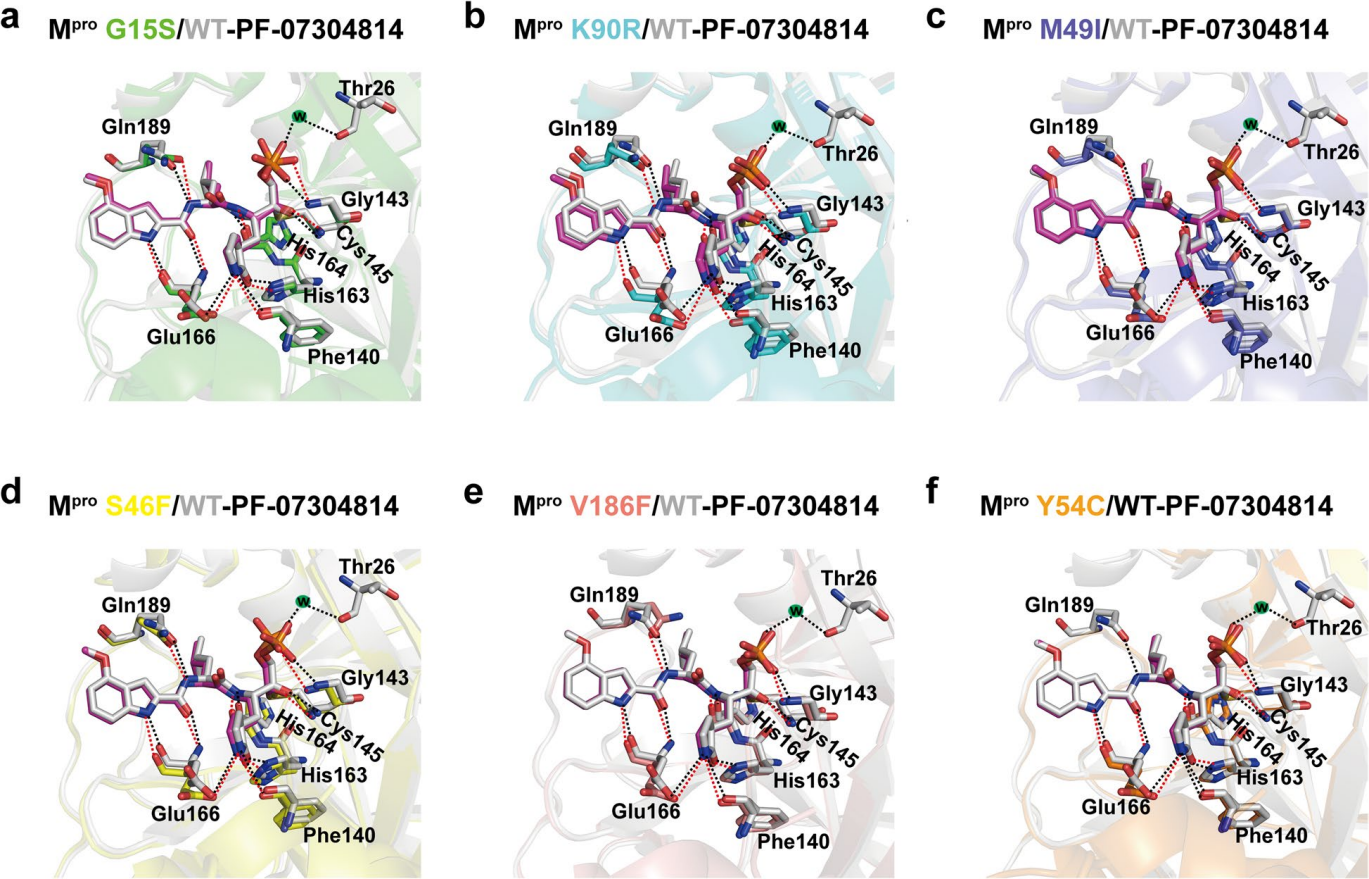
Structural characterization of PF-07304814 bound to SARS-CoV-2 wild-type M^pro^ or M^pro^ mutants. **a-f** Superposition of the x-ray crystal structures of PF-07304814 bound to wild-type (WT) SARS-CoV-2 M^pro^ (**a**-**f**, gray) and SARS-CoV-2 M^pro^ G15S (**a**, green), K90R (**b**, cyan), M49I (**c**, slateblue), S46F (**d**, yellow), V186F (**e**, salmon), and Y54C (**f**, orange). PF-07304814 molecules bound to WT M^pro^ are displayed as gray sticks, while PF-07304814 molecules bound to mutant M^pro^s are shown as magentas sticks. Hydrogen bonds are shown as black dashed lines between WT M^pro^ protein and the inhibitor, while hydrogen bonds are shown as red dashed lines in M^pro^ mutants-PF-07304814 complexes

We further analyzed the interaction details between PF-07304814 and different M^pro^ mutants. PF-07304814 is composed of four moieties, including P1′, P1, P2, and P3 (Fig. 1), and each substituent occupies S1’, S1, S2, and S3 pockets of SARS-CoV-2 M^pro^, respectively. Similar with that in wild-type M^pro^-PF-07304814 interface [23], a C-S covalent bond is formed between the HMK warhead of PF-07304814 and the active-site cysteine (Cys145) in M^pro^ mutants and a tetrahedral carbinol complex is generated. In addition, multiple hydrogen-bonding interactions are formed between PF-07304814 and the residues in the active site of M^pro^ mutants (Fig. 5). A phosphate moiety is presented at the P1′ site of PF-07304814. One of the hydroxyl groups forms a hydrogen bond with the Gly143 backbone NH of mutant M^pro^s, while the carbinol hydroxyl forms an additional hydrogen-bonding interaction with the backbone NH of Cys145 (Fig. 5). These interactions are also observed in the wild-type M^pro^-PF-07304814 interface. However, another hydroxyl group of the phosphate moiety of PF-07304814 forms a water molecule mediated hydrogen bond with Thr26 when bound to wild-type M^pro^ [20]. Such an interaction can not be seen in the M^pro^ mutants-PF-07304814 complexes. The S1 pocket of SARS-CoV-2 M^pro^ has a strong preference for Gln at the P1 position. Upon binding to the wild-type M^pro^ or mutant M^pro^s, the lactam ring displayed at the P1 position of PF-07304814 forms hydrogen-bonding interactions with the Nε2 of H163, the backbone oxygen of Phe140, and the side-chain oxygen of Glu166. This moiety of the inhibitor is expected to mimic Gln at the P1 position of the substrate. Moreover, a hydrogen-bonding interaction can be seen between the P1 NH in PF-07304814 and the main-chain carbonyl oxygen of His164 in wild-type and mutant M^pro^s. A leucine moiety is presented at the P2 position of PF-07304814, while an indole group is displayed at the P3 position. The P2 NH forms a hydrogen-bonding interaction with the side chain of Gln189 in wild-type and mutant M^pro^s (except Y54C). In M^pro^ Y54C-PF-07304814 complex, the hydrogen bond between the Gln189 of mutant M^pro^ and the inhibitor can not be observed. For both wild-type and mutant M^pro^s, the P3 indole of PF-07304814 forms two another hydrogen-bonding interactions with the backbone carbonyl and NH of Glu166. These structural details established that how lufotrelvir recognizes different SARS-CoV-2 M^pro^ mutants, which will be helpful in drug resistance monitoring and further drug development.

## Discussion

Effective antiviral drugs are urgently required to combat the public health threat caused by SARS-CoV-2 and its variants. Main protease (M^pro^) serves as a superior target for anti-SARS-CoV-2 drugs. PF-07304814, also named lufotrelvir, is one of the most advanced M^pro^ inhibitors that have entered clinical trials and also the phosphate prodrug of PF-00835231 that could increase its bioavailability [21]. In addition, PF-07304814 exhibits good tolerability, ADME, and safety in rat models and preclinical trials [21]. Even that the phase 1 data of PF-07304814 were not released and the phase 2/3 trial of PF-07304814 was also suspended, such a promising M^pro^ inhibitor provides a valuable scaffold for drug design. In fact, PF-07321332 (nirmatrelvir), the active ingredient in the oral COVID-19 drug PAXLOVID™, was developed by the modification of PF-07304814. Moreover, as a phosphate ester prodrug of PF-00835231, PF-07304814 displays a very similar molecule structure with its active form. This study used structural biology methods to reveal the differences and similarities of PF-07304814 in binding with different M^pro^ mutants and evaluated the possible conformation change of mutant M^pro^s when bound to this inhibitor. Information on structural changes of M^pro^ mutants when bound to PF-07304814 can be useful for understanding how to inhibit mutant M^pro^s with potential drug resistance and for optimizing the inhibition potency of PF-07321332 as well as PF-00835231. Thus, these data will be informative to develop next generation drugs against wild-type SARS-CoV-2 and its variants.

The use of the electronic density to measure the model fit has some limitation in catching all possible problems in the model [26]. A good manner to evaluate how well a subset of atomic coordinates fits the experimental electron density is the Real Space R-factor (RSR) [27]. Another well-established measure of model fit to the experimental data is the real-space correlation coefficient (RSCC) [28]. Moreover, a RSR value less than 0.3 indicates a good agreement between observed and calculated electron densities and a RSCC value greater than 0.8 indicates a good correlation between calculated and observed electron density. In the present study, the RSR and RSSC values for the ligand PF-07304814 range from 0.08 to 0.17 and from 0.87 to 0.93, respectively, thus indicating a good consistency between the model and the density map for the co-crystallized ligands.

As determined in this study, the binding pattern of PF-07304814 is not significantly perturbed by the six single mutations (G15S, K90R, M49I, S46F, V186F, and Y54C), and the ligand largely maintaining the interaction networks observed in the wild-type M^pro^ with only slight differences. One obvious difference is that a water-mediated hydrogen bond with Thr26 can not be found in the interfaces of mutant M^pro^-PF-07304814 complexes. Another obvious difference is that the hydrogen bond between Gln189 from M^pro^ Y54C and PF-07304814 can not be found. A water bridge between the ligand and M^pro^ Thr26 plays an important role in ligand binding [29]. Gln189 of SARS-CoV-2 M^pro^ has also been identified as one of the hot spot residues in the interaction with inhibitors and a hydrogen bonding with Gln189 may enhance the potency of M^pro^ inhibitor [29, 30]. These differences may impact the binding affinities between M^pro^ mutants and PF-07304814.

We then calculate the binding energies between PF-07304814 and the targeted protein to have a glimpse of the potential of PF-07304814 as a ligand to the active site of M^pro^ mutants. Specially, the binding between PF-07304814 and Y54C mutant is much higher than that between PF-07304814 and wild-type M^pro^, which may be due to the lost of hydrogen bonding interaction between the Gln189 in M^pro^ Y54C and the ligand PF-07304814. Other M^pro^ mutants also show higher binding energy values with PF-07304814 compared to wild-type M^pro^, which may be due to the lost of a water meidicated hydrogen bond between Thr26 of M^pro^s. As evidenced in the literature, the covalent docking may not be able to accurately reveal the real binding strength between the ligand and targeted proteins [31], because covalent docking disregards to explicitly explore the reactivity of the covalent inhibitors. Thus, further biochemical assays are needed in the future to evaluate the inhibition efficacy of PF-07304814 against these M^pro^ mutants.

The present study investigated the impact of single amino acid substitutions of M^pro^s on the its interaction with PF-07304814. However, main proteases from the circulating SARS-CoV-2 strains tend to possess multiple mutations. Next steps should also include experimental and structural characterization of the impact of combined mutations on the interactions network between PF-07304814 and SARS-CoV-2 M^pro^. As PF-07304814 is a promising drug candidate to treat COVID-19, M^pro^ mutations that cover a larger variety of SARS-CoV-2 lineages should also be investigated in further study.

## Materials and methods

### Protein expression and purification

Expression of M^pro^ mutants of SARS-CoV-2 (including G15S, S46F, M49I, Y54C, K90R, and V186F) was performed according to previous description [23]. Briefly, pET-28a plasmids containing the entire coding sequences were transformed into competent *Escherichia coli* (*E. coli*) Rosetta DE3 cells. After grown in LB (Luria-Bertani) broth with the OD600 reaching 0.6–0.8 and induced with 500 µmol/L isopropyl-β-d-thiogalactopyranoside (IPTG), the *E. coli* cells started to produce mutant M^pro^ proteins. The cells were then centrifuged at 10,000 × g for 15 min at 4 ℃. The supernatants were discarded, while the cell pellets were collected, resuspended using lysis buffer, and lysed by sonication. The cleared lysate was subjected to immobilized metal affinity chromatography on a HisTrap HP 5 mL column (Cytiva). Imidazole gradient treatment was employed to elute the target proteins. Elution fractions containing mutant M^pro^ proteins were harvested and incubated with TEV protease to remove the N-terminal His-tag. The digested M^pro^s were subjected to gel filtration for further purification. High-purity mutant M^pro^ fractions were collected with centrifuge tubes and concentrated using concentrator tubes with the molecular weight cutoff of 10 kDa (Millipore).

### Crystallization

The eluted SARS-CoV-2 M^pro^ mutants were concentrated to 5 mg/mL, and then incubated with PF-07304814 molecules at a molar ratio of 1:3 on ice for 30 min. Crystallization was carried out at 18 °C via the sitting drop vapor diffusion technique. After several days, the crystals of six SARS-CoV-2 M^pro^ mutants in complex with PF-07304814 were obtained. The final crystallization condition of the M^pro^ G15S-PF-07304814 complex was 0.15 M Tris pH 8.0, 30% (w/v) PEG 4000. The final crystallization condition of the M^pro^ S46F-07304814 complex was 0.20 M Na_2_SO_4_, 24% (w/v) PEG 3350. The final crystallization condition of the M^pro^ M49I-07304814 complex was 0.15 M Na_2_SO_4_, 20% (w/v) PEG 3350. The final crystallization condition of the M^pro^ Y54C-07304814 complex was 0.1 M BIS-Tris pH 6.5, 20% (w/v) PEG 3350. The final crystallization condition of the M^pro^ K90R-07304814 complex was 0.15 M Na_2_SO_4_, 22% (w/v) PEG 3350. The final crystallization condition of the M^pro^ V186F-07304814 complex was 0.16 M Na_2_SO_4_, 20% (w/v) PEG 3350.

### Data Collection, structure determination, and refinement

Before X-ray diffraction data collection, the crystals of SARS-CoV-2 M^pro^ mutants in complex with PF-07304814 were soaked in the crystallization buffer consisting of 20% glycerol and then flash cooled in liquid nitrogen. X-ray diffraction data were collected at 100 K at BL10U2 of the Shanghai Synchrotron Radiation Facility (SSRF). All the data sets were processed using HKL2000 software [32]. All the complex structures were determined via the molecular replacement method by using the Phaser program [33]. Coot and Phenix softwares were used for atomic model building and maximum likelihood-based refinement [34, 35]. The stereochemical qualities of the final models were assessed with MolProbity [36]. The data collection and processing statistics and structural refinement statistics for the M^pro^ mutants-PF-07304814 complexes are shown in Table 1. The values of real-space R-factor (RSR) and real-space correlation coefficient (RSCC) were calculated to measure the electron density fit for PF-07304814 molecules in the complex structures [27, 28].

### Molecular docking analysis

The co-crystallized M^pro^ mutants and the ligands were prepared in advance of docking. For M^pro^ mutants, the chain B of protein was kept and other atoms were deleted. For the ligand PF-07304814, the residue Cys145 (atoms SG, CA, CB, and C) where Michael addition reaction took place was appended to guide covalent docking. Then, the AutoDockTools-1.5.7 program was employed to add polar hydrogen atoms and assign Kollman charges. The grid box defining the binding pocket was set as the center of the ligand with length of 30 angstrom to all three dimensions. Finally, the ADFR program was applied to generate the affinity maps and perform covalent docking [37].

## Data Availability

Coordinates and structure factors for SARS-CoV-2 M^pro^ G15S, S46F, and M49I in complex with PF-07304814 have been deposited in PDB under accession code 8HVU, 8HVV, and 8HVW, respectively. Coordinates and structure factors for SARS-CoV-2 M^pro^ Y54C, K90R, and V186F in complex with PF-07304814 have been deposited in PDB under accession code 8HVX, 8HVY, and 8HVZ, respectively.
